# P03, the microfocus and nanofocus X-ray scattering (MiNaXS) beamline of the PETRA III storage ring: the microfocus endstation

**DOI:** 10.1107/S0909049512016895

**Published:** 2012-05-10

**Authors:** Adeline Buffet, André Rothkirch, Ralph Döhrmann, Volker Körstgens, Mottakin M. Abul Kashem, Jan Perlich, Gerd Herzog, Matthias Schwartzkopf, Rainer Gehrke, Peter Müller-Buschbaum, Stephan V. Roth

**Affiliations:** a DESY, Notkestrasse 85, 22603 Hamburg, Germany; b TU München, Physik-Department, Lehrstuhl für Funktionelle Materialien, James-Franck-Strasse 1, 85748 Garching, Germany

**Keywords:** X-ray scattering, microfocus, kinetic studies, nanocomposites

## Abstract

The P03 beamline, also called the microfocus and nanofocus X-ray scattering (MiNaXS) beamline, exploits the excellent photon beam properties of the low-emittance source PETRA III to provide a microfocused/nanofocused beam with ultra-high intensity for time-resolved X-ray scattering experiments. The beamline has been designed to perform X-ray scattering in both transmission and reflection geometries. The microfocus endstation started user operation in May 2011 ▶. An overview of the beamline status and of some representative results highlighting the performance of the microfocus endstation at MiNaXS are given.

## Abstract 2

The MiNaXS (P03) beamline of the new third-generation synchrotron radiation source PETRA III (DESY, Germany) has been designed to perform small-, ultra-small- and wide-angle X-ray scattering in both transmission and grazing-incidence geometries. The high photon flux available at the beamline enables time-resolved investigations of kinetic phenomena with a time resolution below 100 ms. The microfocus endstation started user operation in May 2011.

## 1. Introduction

The new third-generation 6 GeV synchrotron facility PETRA III (Franz *et al.*, 2006 ▶) operates in a top-up injection mode at a positron beam current of 100 mA. Among the 14 new beamlines, the P03 beamline, also called the microfocus and nanofocus X-ray scattering (MiNaXS) beamline, was designed to satisfy the needs of a large variety of X-ray scattering experiments (Riekel, 2000 ▶; Müller-Buschbaum *et al.*, 2007 ▶; Sasaki *et al.*, 2007 ▶). The MiNaXS beamline has been optimized for performing small-, ultra-small- and wide-angle X-ray scattering in both transmission and grazing-incidence geometries. Owing to the excellent photon beam properties of the low-emittance source PETRA III, the photon flux available at MiNaXS allows for brilliance-demanding investigations such as *in situ* time-resolved experiments (time resolution of the order of a few milliseconds). The beamline consists of three hutches: the optics hutch and two experiment hutches, also called the endstations. One is dedicated to microfocus X-ray scattering experiments, whereas the other allows for complementary investigations using a nanofocus beam (Krywka *et al.*, 2010 ▶, 2012 ▶). In this article we describe the beamline optics and provide an overview of the instrumentation available in the microfocus endstation which is already in user operation. Finally, selected results are presented to illustrate the performance of the beamline.

## 2. Beamline optics

The beamline is supplied with photons from a 2 m-long U29 undulator located in a high-β section of the PETRA III storage ring. The size and divergence (r.m.s. values) of the source, for high-β sections at PETRA III, are 140 µm × 5.6 µm and 7.9 µrad × 4.1 µrad, respectively (Barthelmess *et al.*, 2008 ▶). A high-β undulator is a prerequisite to combining low divergence and high-flux microfocus beam. This choice enables high-speed *in situ* scattering experiments with high spatial resolution (see §4).

Monochromatic beam is delivered by a large-offset fixed-exit monochromator located in the optics hutch. There are two main reasons for using a large downward-offset monochromator. First, there is only 5 mrad horizontal angle between the canted undulator beamlines P02 and P03. The compact arrangement of the two beamlines can be achieved by shifting one of the two beamlines in the vertical direction. This is done by using a so-called large-offset monochromator at the P03 beamline (Horbach *et al.*, 2010 ▶). The second issue is the stability of the installation, especially when the photon beam extent is within a range of a few square micrometers. By having an X-ray beam at a height of 900 mm above the floor (instead of 1400 mm) in the endstations, we significantly reduce the amplitudes of potential (mechanical) vibrations.

**Figure 1:**
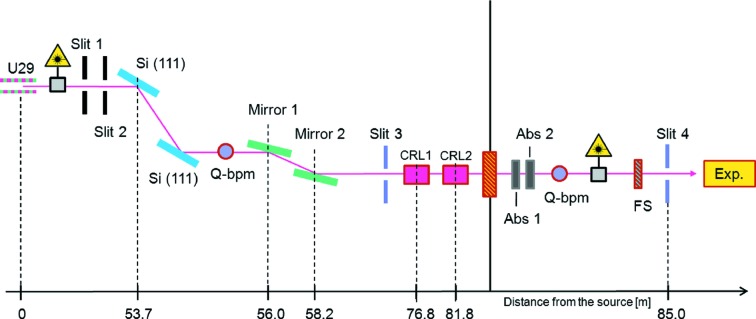
Sketch of the MiNaXS beamline optics. FS stands for fast shutter, Abs for Absorber, CRL for compound refractive lens, Q-bpm for quad beam position monitor and Exp for experiment. The distance from the undulator source is indicated in meters. Two lasers are available at the beamline. They are used for pre-alignment of the monochromator and the sample, respectively.

The monochromator is equipped with a pair of cryocooled silicon (111) crystals having a vertical offset of 490 mm downward. The energy can be varied from 8 to 23 keV with a resolution of Δ*E*/*E* ≃ 10^−4^. A scheme of the main optic components of the beamline is given in Fig. 1 ▶. In addition, a pair of multilayers is under design. The latter option will be especially useful for nanofocus and µUSAXS applications owing to the higher flux provided by the pink beam (Tsuruta *et al.*, 1998 ▶; Hexemer *et al.*, 2006 ▶). In this latter configuration an energy resolution of Δ*E*/*E* ≃ 0.5 × 10^−2^ is expected at the fixed energies of 10 keV and 13 keV.

**Figure 2:**
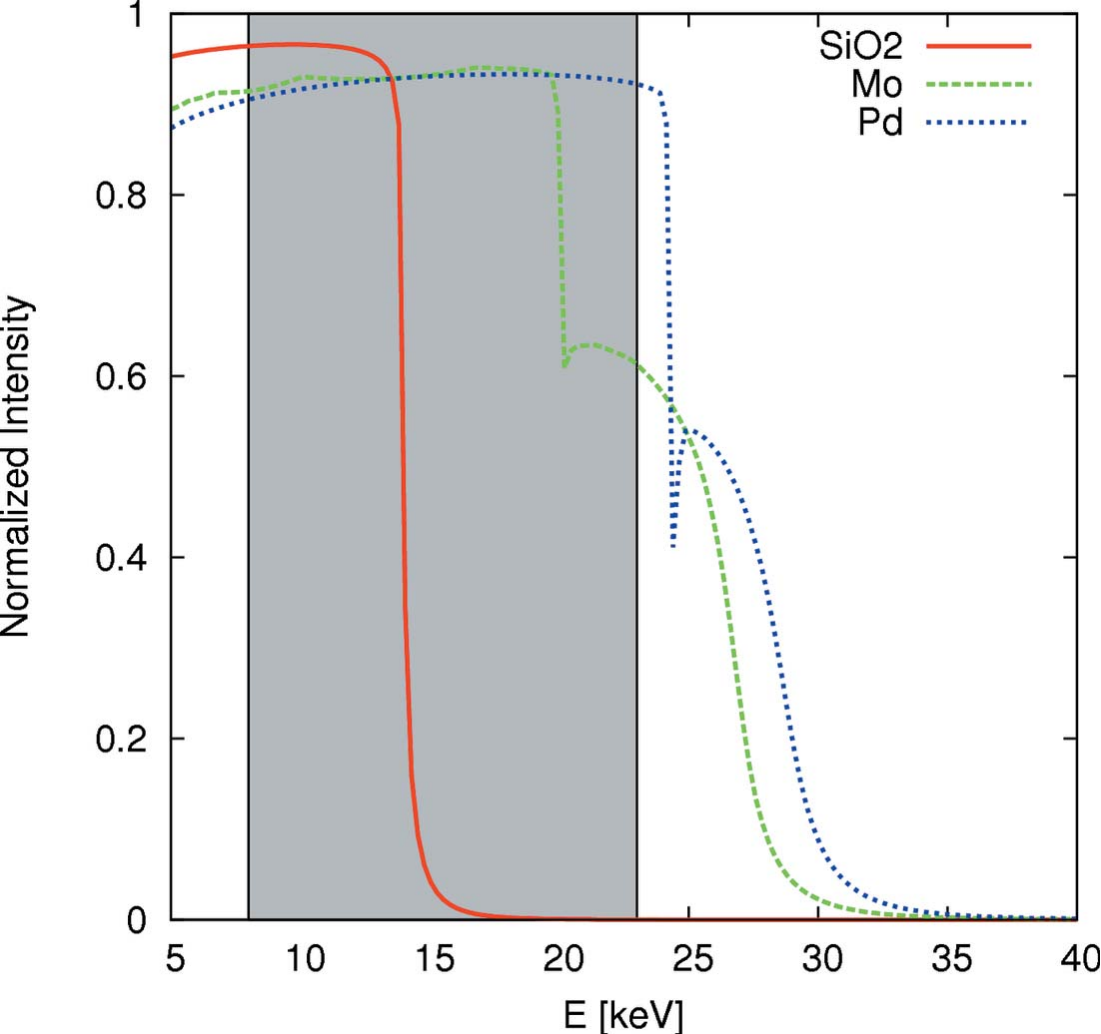
Calculated normalized intensity after the second mirror when using SiO_2_ coating (red continuous line), Mo coating (green dashed line) or Pd coating (blue dotted line). The X-ray beam is reflected by the mirrors under a grazing incident angle of 2.27 mrad. The gray box indicates the X-ray energy range provided by the large offset monochromator at P03.

A planar double mirror (reflection angle = 2.27 mrad, vertical offset = 10 mm downward) located in the optics hutch past the monochromator is used to suppress higher harmonics. Three different coatings are available: a SiO_2_ coating for the energy range 8–12 keV, a molybdenum (Mo) coating for the energy range 12–18 keV, and a palladium (Pd) coating for the energy range 18–23 keV. The theoretical curves in Fig. 2 ▶ show the calculated suppression of higher harmonics.

At the MiNaXS beamline, beryllium compound refractive lenses (CRLs) are used for both collimation and focusing of the beam (Lengeler *et al.*, 2005 ▶). Two transfocators are available at respective distances of 76.8 m and 81.8 m from the source. They are situated between the optics hutch and the microfocus endstation (CRL1 and CRL2 in Fig. 1 ▶). This allows beam size and divergence to be optimized according to the experiment.

**Figure 3:**
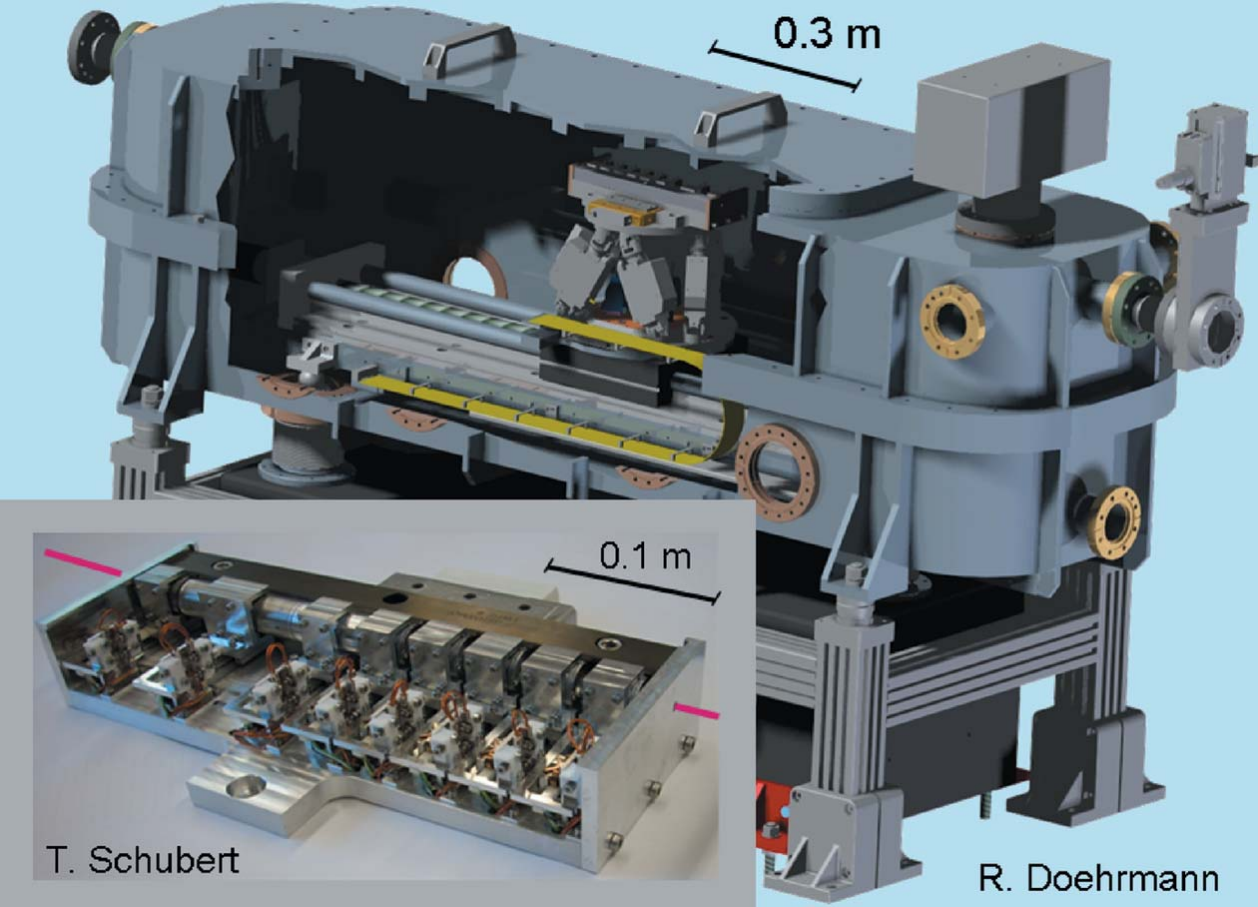
Drawing of a transfocator chamber and photograph of a transfocator at P03. The transfocator consists of eight blocks of Be lenses which can be moved either in or out of the monochromatic X-ray beam.

**Figure 4:**
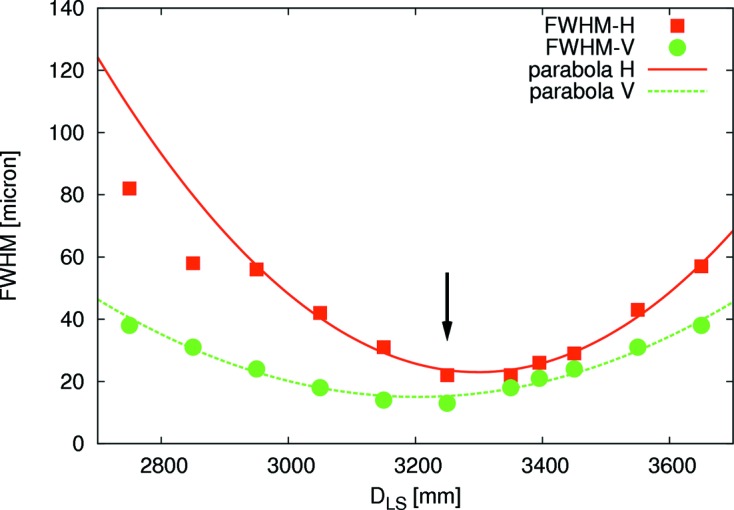
Beam size as a function of the lens-to-sample distance *D*_LS_ (*N* = 16 CRLs): horizontal FWHM (red squares), vertical FWHM (green circles). The error bars are shown below the symbol size. A parabolic approximation of the data is indicated by the lines. It can be used to determine the optimal focus position in the vertical (green dashed line) and horizontal (red solid line) directions.

Each transfocator consists of eight blocks of CRLs which can be moved in/out independently of the X-ray beam path. Each lens has an apex curvature radius of 200 µm and the entire optical system results in an acceptance of 500 µm in beam diameter. Currently, a total of 25 CRLs (CRL1) and 56 CRLs (CRL2) provides a flexible system to meet various experiment needs. Each transfocator is mounted on a hexapod (M-824, PI, Germany) that can be translated along an axis parallel to the X-ray beam as shown in Fig. 3 ▶. The number of CRLs in the beam as well as the position of the transfocator can be adjusted such that the focus coincides with the sample or detector position for a given X-ray energy. For instance, at an energy of 12.8 ± 0.1 keV, a beam size (H × V) of 23 (±1) µm × 13 (±1) µm can be achieved at the sample position by using 16 CRLs of the second transfocator (*N*_2_ = 16) and a lens-to-sample distance *D*_LS_ of 3.25 m (see also Fig. 4 ▶). The measured beam size is in good agreement with the beam size obtained by ray-tracing simulations of the full beamline using the software package *SHADOW* (Welnak *et al.*, 1994 ▶). The previously described optical configuration (microfocus beam) provides a photon flux of 5 (±1) × 10^11^ photons s^−1^ at the sample position. The absolute photon flux was measured using a calibrated polyethylene (LUPOLEN) standard (Endres *et al.*, 1997 ▶) at an energy of 12.8 ± 0.1 keV and using the third harmonic of the undulator source. All numbers are given for the PETRA III storage ring operating at 100 mA.

Four slit systems are available at the beamline. The first two slit systems are located between the undulator and the monochromator selecting the relevant beam area originating from the undulator source and thus reducing the heat load on the first crystal of the monochromator. The third slit system is located before the CRLs for optimizing the monochromatic X-ray beam size according to the lens acceptance. The fourth slit system, also called the guard slit system, is mounted close ahead of the sample blocking parasitic scattering. The third and fourth slit systems consist of four tantalum blades each being individually driven by piezo motors. The space in between the two transfocators is planned to house an additional slit system that can be used as a secondary source after focusing using CRL1.

Two SESO X-ray beam monitors (Société Européenne de Systèmes Optiques, France) can be used to optimize the alignment of the CRLs and of the slits: a low-resolution SESO monitor with a pixel size of 7.4 µm × 7.4 µm and a field of view of 5.8 mm × 4.9 mm, and a high-resolution SESO monitor with a pixel size of 0.7 µm × 0.7 µm and a field of view of 0.6 mm × 0.5 mm.^1^

Pre-alignment of the monochromator and of the sample can be performed using diode lasers (51nanoFCM, Schäfter and Kirchhoff GmbH; see Fig. 1 ▶). By this, a preliminary sample adjustment can easily be made with an accuracy of the order of 1 mm. More accurate sample alignment is performed in a second step using the X-ray beam and a diode beamstop or a two-dimensional detector. This procedure allows an accuracy in sample alignment down to about 1 µm to be achieved. Different combinations of absorbers (Abs1 and Abs2 in Fig. 1 ▶) can be used to prevent sample damage. A large set of aluminium (0.1 to 1 mm) and silver (0.0125 to 1.5 mm) foils are available to reduce the beam intensity at the sample position.

Finally, two quad beam position monitors (Q-bpm, connected to I404 digital electrometer; FMB Oxford, UK) are used for permanent monitoring of the beam position and intensity. They are located in the optics hutch past the monochromator and in the microfocus endstation behind the absorbers, respectively.

## 3. Microfocus endstation

Several sample environments are available at the beamline. To name just a few, a droplet deposition apparatus, a microfluidic cell adapted for GISAXS (grazing-incidence small-angle X-ray scattering) investigation (Moulin *et al.*, 2008 ▶; Metwalli *et al.*, 2009 ▶), a sputter deposition chamber and an imaging ellipsometer (Körstgens *et al.*, 2010 ▶; Roth *et al.*, 2011 ▶). Sample environments provided by the users can be implemented either on a hexapod (M-824, PI, Germany) or on a HUBER goniometer. Whereas the hexapod allows mountings up to a weight of 10 kg, the goniometer is foreseen for heavier loads up to 180 kg.

**Figure 5:**
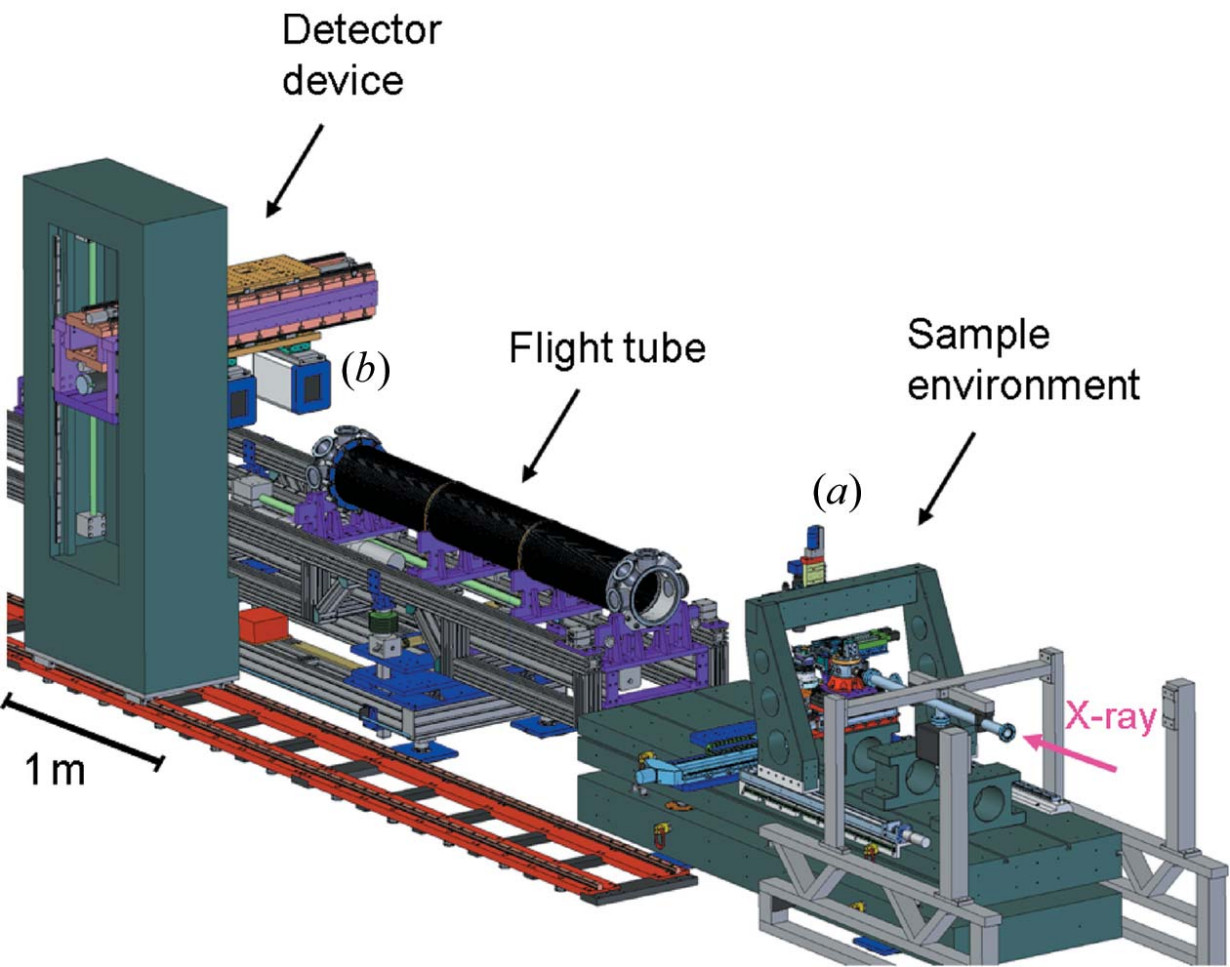
Illustration of the microfocus endstation at MiNaXS: standard sample environment. The adaptive flight tube, detector device, optical microscope (*a*), and Pilatus 300k detector (*b*) are indicated.

An optical microscope (Keyence) equipped with a long-working-distance objective (VH-Z50L) in combination with a CCD camera allows for remote-controlled live imaging (up to 90 frames s^−1^) of the sample at the micrometer scale while performing an experiment [see (*a*) in Fig. 5 ▶]. The system enables high-magnification (50× to 500× optical zoom) observation while maintaining a long working distance of 85 mm. The objective is mounted on an *xyz* stage and can be tilted from −45 to 45° (0° corresponds to an observation from top). Two CCD cameras (Allied Vision Technologies, Germany) are available: a Prosilica GC650C (659 pixels × 493 pixels, full resolution frame rate of 90 frames s^−1^) and a Prosilica GC1380 (1360 pixels × 1024 pixels, full resolution frame rate of 20.2 frames s^−1^) for high-speed and high-resolution optical imaging, respectively.

**Figure 6:**
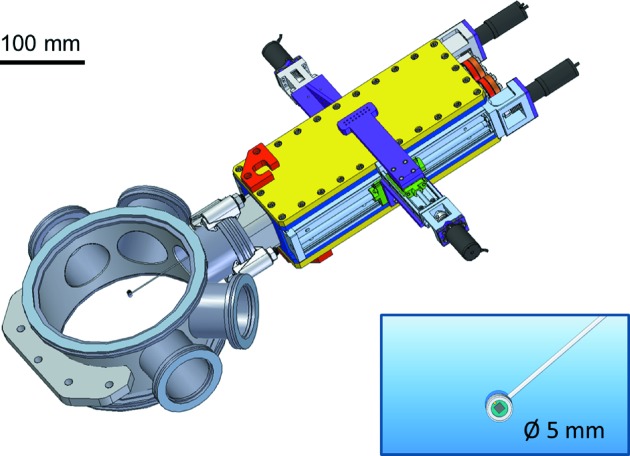
Drawing of the in-vacuum beamstop device. Zoom-in: drawing of a 5 mm-diameter diode beamstop.

An evacuated flight tube (sealed by kapton windows) is placed between the sample and the detector to reduce additional signal due to air scattering. The sample-to-detector distance (*D*_SD_) can be easily changed owing to the adaptive flight tube device and the moving detector table (see Fig. 5 ▶). Changing of the flight tube length is carried out in a few minutes without breaking vacuum. Presently, sample-to-detector distances *D*_SD_ from 60 cm to 5 m are possible. This allows 2θ acceptance angles ranging from 0.8 to ≥6° to be typically achieved. Longer sample-to-detector distances up to 10 m are foreseen for µUSAXS and µGIUSAXS measurements. An in-vacuum beamstop device was recently implemented. It allows for the use of two modular in-vacuum beamstops, being equipped with a diode for normalization of the X-ray scattering data. An illustration is shown in Fig. 6 ▶.

Currently, three detectors are available at the MiNaXS beamline. A CCD detector Quad-RO 4096^2^ can be used for high-spatial-resolution measurements (pixel size = 15 µm × 15 µm). A single-photon-counting detector, Pilatus 300k from DECTRIS (Switzerland)^3^, allows for fast measurements with readout times down to 3 ms (pixel size = 172 µm × 172 µm). A silicon drift X-ray detector spectrometer, fluorescence Vortex detector from SII Nanotechnology^4^, was recently implemented for simultaneous X-ray scattering and X-ray fluorescence measurements (50 mm^2^ active area). Additional detectors can be borrowed from the DESY detector loan pool^5^ and installed at the beamline, for instance a Pilatus 1M detector.

A video camera server (CCTV with up to 16 cameras, currently 7) allows for live monitoring of the experiment area and more particularly of the sample environment. This is very useful in the case of complex sample environments.

To ease the processing of the collected data, the first version of an online data analysis software is available at the beamline. It is currently under development in close collaboration with the user community. It enables preliminary online analysis of large data sets resulting from, for example, high-throughput X-ray scattering experiments using area detectors. The details, including the code, are currently being prepared for publication.

## 4. Highlights

The MiNaXS beamline enables novel (GI)SAXS and (GI)WAXS investigations in many areas of research, such as polymer science, hybrid nanocomposite nanotechnology, microfluidics or thin-film physics. The MiNaXS beamline is optimized to deliver a microfocused X-ray beam with a low divergence and a high photon flux at the sample position to allow for kinetic investigations in confined geometry with high time resolution.

**Figure 7:**
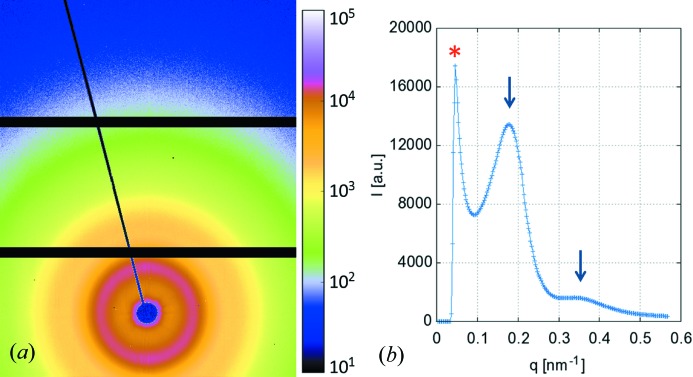
(*a*) Two-dimensional µSAXS data of a polyethylene (LUPOLEN) calibration sample. (*b*) Azimuthal integrated intensity. The vertical arrows highlight the positions of the first-order and second-order maxima of the LUPOLEN calibration sample. The SAXS resolution is imposed by the beamstop size as indicated by the star (*) at *q* = 0.05 ± 0.01  nm^−1^.

**Figure 8:**
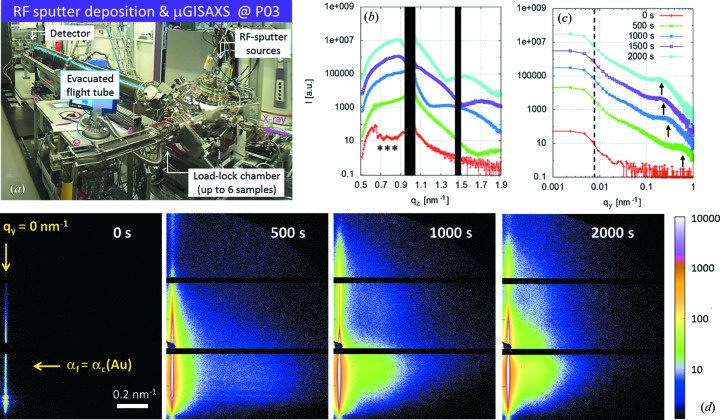
(*a*) Photograph of the RF sputter deposition experiment set-up at MiNaXS. (*b*) Detector cut *I*[*q*_*z*_(*t*)] at *q*_*y*_ = 0 nm^−1^. The black areas correspond to the shadow of the specular beamstop and to the gaps in between modules of the Pilatus 300k detector. The curves are shifted for clarity. Resonant diffuse scattering stemming from the thin polymer film is indicated by the stars (***). (*c*) Out-of-plane cut *I*[*q*_*y*_(*t*)] at α_f_ = α_c_(Au). The curves are shifted for clarity. (*d*) µGISAXS data recorded at the MiNaXS beamline at an X-ray energy of 13.0 ± 0.1 keV, an incident angle of 0.435 ± 0.005°, a sample-to-detector distance of 4823 ± 2 mm and with an exposure time of 95 ms (exposure period 100 ms). The arrows indicate the positions of the detector (vertical arrow) and out-of-plane cuts (horizontal arrow) shown in (*b*) and (*c*), respectively. In this configuration the resolution limit is imposed by the beam divergence as indicated by the vertical dashed line at *q*_*y*_ = 0.005 ± 0.001 nm^−1^ in (*c*).

For instance, in the rapidly growing research field of microfluidics, characterization tools compatible with the miniaturization of the devices are required. Practically, the X-ray beam size has to be optimized to allow for scanning inside flow channels, which are typically of the order of some 10 to 100 µm (Otten *et al.*, 2005 ▶; Barett *et al.*, 2006 ▶; Rondeau & Cooper-White, 2008 ▶). For high-resolution SAXS, it is not only important to have a small beam size but also determining to have a small beam divergence (Riekel *et al.*, 2000 ▶; Müller-Buschbaum, 2003 ▶). Indeed, in the last few years, a new demand for a probe that is capable of characterizing the morphology of the sample on both the nanometer and the micrometer scale has been arising, in particular in the research field of nanocomposites (Li *et al.*, 2011 ▶). For a given sample-to-detector distance and a given X-ray energy the SAXS resolution is limited to a maximum resolvable length scale defined by the X-ray beam divergence and the geometrical limits of the transmission set-up imposed by the beamstop size and the detector pixel size. The lower the beam divergence, the larger the maximum resolvable length scale. At the MiNaXS beamline the resolution in transmission geometry is imposed by the beamstop size, as shown in Fig. 7 ▶. For instance, at a photon energy of 13 keV and a sample-to-detector distance of 4.8 m the SAXS resolution is currently about 130 nm, whereas, in grazing-incidence geometry, structures up to 1 µm can be resolved (see Fig. 8*c* ▶). Actually, in this latter configuration, the resolution is only limited by the beam divergence. Finally, high photon flux at the sample position is crucial for performing time-resolved investigations of dynamic processes such as chemical or biochemical reactions (Martel *et al.*, 2008 ▶), nanoparticle assembly processes (Yang *et al.*, 2009 ▶), growth kinetics (Renaud *et al.*, 2003 ▶) and transport phenomena (Bauer *et al.*, 2006 ▶; Kim *et al.*, 2007 ▶).

Out of the wide variety of experiments already performed at the beamline, we chose two examples to demonstrate the performance of the beamline. The first example highlights the potential capabilities of the beamline in terms of time-resolved investigation of kinetic processes. In the second example we used the unique combination of imaging ellipsometry and µGISAXS to investigate droplet solution casting of gold nanoparticles on a polymer template.

In both cases we used a microfocus photon beam of 35 µm × 22 µm (H × V) at the sample position. The microfocus beam was obtained by using 12 CRLs of the second transfocator (see Fig. 1 ▶). The focusing of the beam onto the sample position results in high real-space resolution at the position of the sample and thus allows for the investigation of local structures. In addition, the possibility of combining microfocus beam GISAXS (µGISAXS) (Müller-Buschbaum *et al.*, 2007 ▶) with scanning of the sample with respect to the X-ray beam is of particular interest especially in the case of a non-homogeneous specimen. In this context we give as an example the case of droplet solution casting where structural gradients can be observed from the center towards the rim of the droplet (see also Roth *et al.*, 2010 ▶). In the sputter deposition study presented in §4.1 the sample size is limited by the RF-source diameter (1"). By reducing the beam footprint (microfocus beam) on the specimen in grazing incidence, we can achieve a high signal-to-noise ratio at a time resolution down to the millisecond regime.

### 4.1. *In situ* and real-time GISAXS investigation during sputter deposition of metal on a polymer template

The combination of high photon flux and detectors with low readout times offers the opportunity to investigate kinetics with high time resolution in the millisecond regime. This is of special interest in research fields dealing with the understanding of growth process kinetics (Renaud *et al.*, 2003 ▶; Kaune *et al.*, 2009 ▶; Buffet *et al.*, 2011 ▶) or transport phenomena (Thiele *et al.*, 2010 ▶). Among the technologically relevant materials are metal–polymer nanocomposites, which allow for the combination of the mechanical (flexibility) or thermal properties of polymers with the electronic or magnetic properties of metals (Schürmann *et al.*, 2006 ▶; Faupel *et al.*, 2010 ▶). In this field, characterization of the nanocomposite nanostructure and comprehension of the nanocomposite growth kinetics are currently undertaken at the MiNaXS beamline with a time resolution of 100 ms.

Sample images illustrating the excellent signal quality obtained at the frame rate of 10 frames s^−1^ are given in Fig. 8(*d*) ▶. The images show µGISAXS data at selected points in time of the growth process of a gold (Au) metallic layer on top of a polymer template. The µGISAXS measurements were performed at an X-ray energy *E* = 13.0 ± 0.1 keV and an incident angle α_i_ = 0.43 ± 0.005° (above the critical angle of all materials involved: Si, PS and Au). The two-dimensional data were recorded using a Pilatus 300k (DECTRIS) detector (exposure time of 95 ms, exposure period of 100 ms). The sample-to-detector distance was *D*_SD_ = 4.82 m resulting in a *q*_*y*_ range from 0.008 to 1 nm^−1^.

For the experiment, a new inhouse-developed RF-sputter deposition chamber was implemented at the MiNaXS beamline. A photograph of the experiment set-up is shown in Fig. 8(*a*) ▶. Before the start of sputter deposition (*t* = 0 s), resonant diffuse scattering indicating correlated roughness of the homopolymer thin film template (Holý & Baumbach, 1994 ▶; Müller-Buschbaum & Stamm, 1998 ▶) is observed within the short exposure time of 95 ms, as indicated by the stars in Fig. 8(*b*) ▶. Analysis of the scattering data highlights two phases in the growth process of the metallic layer. In the first phase the emergence of side maxima and the shift of those maxima toward lower *q*_*y*_ values indicate the lateral growth of Au nanoclusters [vertical arrows in Fig. 8(*c*) ▶]. In the second phase the increase in the number of maxima in the detector cut and the shift of those maxima toward smaller *q*_*z*_ values is a signature of the vertical growth of a thin metallic layer (Fig. 8*b* ▶). The two-dimensional µGISAXS data shown in Fig. 8 ▶ clearly demonstrate the capability to achieve remarkable signal quality at a short exposure time and the high potential of the MiNaXS beamline for experiments demanding exceptional time resolution.

### 4.2. Combining imaging ellipsometry and µGISAXS

An *in situ* study of metal–polymer nanocomposite formation was performed at the MiNaXS beamline by using the unique combination of imaging ellipsometry and µGISAXS. Given the high photon flux available at the beamline, we were able to investigate *in situ* the ordering and clustering of the metallic nanoparticles deposited on top of a polymer template.

**Figure 9:**
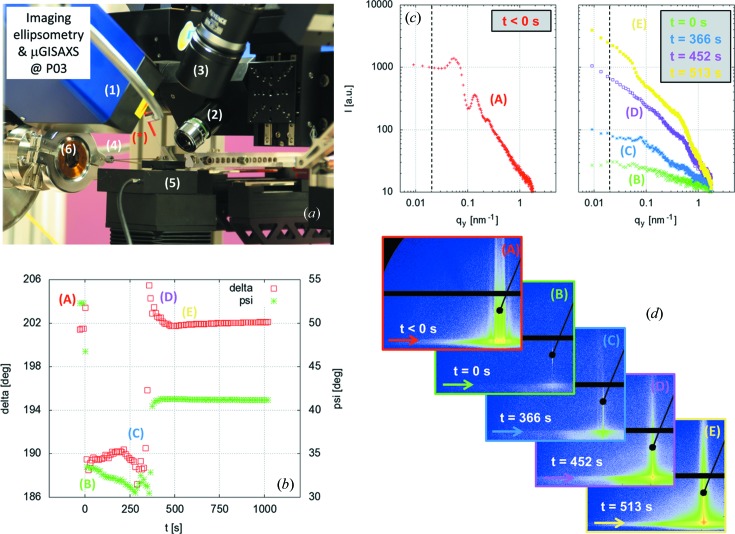
Combined µGISAXS and imaging ellipsometry at MiNaXS. (*a*) Photograph of the experiment set-up (sample environment): (*) needle, highlighted by the red line; (1) ellipsometer laser arm; (2) ellipsometer detector arm; (3) optical microscope; (4) diode beamstop; (5) sample stage; (6) flight tube entrance window. (*b*) Ellipsometer data: delta (Δ) and psi (Ψ) as a function of time. (*c*) Out-of-plane cuts *I*[*q*_*y*_(*t*)] at α_f_ = α_c_(Si) as indicated by the arrows in the two-dimensional GISAXS data shown in (*d*): (*A*) *t* < 0 s, (*B*) *t* = 0 s, (*C*) *t* = 366 s, (*D*) *t* = 452 s and (*E*) *t* = 513 s. The time that a droplet of the gold nanoparticle solution was deposited onto the polymer template defines *t* = 0 s.

For the experiment, a custom-designed scanning probe ellipsometric microscope including an imaging ellipsometer, a single-wavelength (532 nm) instrument from Accurion (Nanofilm Technologie GmbH), was installed at the MiNaXS beamline on the heavy-load goniometer stage, as shown in Fig. 9(*a*) ▶. A single calibration of the instrument is necessary to ensure that the ellipsometer laser beam and the X-ray beam cross each other at one known point on the sample surface (Körstgens *et al.*, 2010 ▶).

Gold (Au) nanoparticles in aqueous solution were deposited on top of a carboxylated polystyrene (PS) colloidal template by solution casting. The nominal diameters of the Au nanoparticles (Nanocs, USA) and of the carboxylated PS colloids (Kisker, Germany) were 20 nm and 100 nm, respectively. The droplets had a nominal volume of 33 ± 8 µL. The PS colloidal film was prepared on a base-cleaned silicon (Si) substrate by spin-coating. A complete description of the polymer template preparation is given by Roth *et al.* (2011 ▶).

The µGISAXS measurements were performed at an X-ray energy *E* = 12.8 ± 0.1 keV and an incident angle α_i_ = 0.45° (above the critical angle of all materials involved: Si, PS and Au). The two-dimensional data were recorded using a Pilatus 300k (DECTRIS) detector (typical exposure time 5 s). The sample-to-detector distance was *D*_SD_ = 2.74 m resulting in a *q*_*y*_ range from 0.02 to 2 nm^−1^ for this experiment.

Imaging ellipsometry data and µGISAXS data are shown in Figs. 9(*b*)–9(*d*) ▶. The deposition of a droplet of the solution onto the polymer template defines the time *t* = 0 s. The time-resolved evolution of the sample nanostructure lateral ordering can be followed from the out-of-plane cuts (Fig. 9*c* ▶). Before deposition, *t* < 0 s (*A*), well pronounced intensity maxima are observed. Such maxima are characteristic of the PS colloidal template. With the droplet deposition at time *t* = 0 s (*B*), a strong decrease in the scattering intensity together with a sudden drop in the ellipsometric delta (Δ) and psi (Ψ) values are observed. This is due to the strong absorption of the X-ray beam by the droplet. For *t* > 0 s, while solvent evaporation induced changes in Δ and Ψ are *in situ* monitored by imaging ellipsometry, lateral rearrangement of the PS colloids and of the Au nanoparticles is observed in the GISAXS data with the emergence of a shoulder-like peak in the out-of-plane cuts (*C*, *D*, *E*). The build-up of the Au nanoparticle layer and the morphological rearrangement of the PS layer can be described by a model based on four distinct time regimes. A detailed analysis can be found by Roth *et al.* (2011 ▶).

## 5. Summary and conclusion

After a successful beamline commissioning phase and very promising friendly user experiments, the MiNaXS beamline (P03) of the PETRA III storage ring is now in operation. Within the last six months the microfocus endstation at MiNaXS has already shown to be a versatile instrument for X-ray scattering from soft matter to hard matter surfaces and thin films, but also for various scattering geometries such as SAXS, WAXS, GISAXS and GIWAXS. Exploiting the excellent photon beam properties of the low-emittance source PETRA III, a micro-focused (H × V ≥ 23 µm × 13 µm) monochromatic X-ray beam with high intensity (≥10^11^ photons s^−1^) can be provided in the energy range 8–23 keV. The possibility to change the sample-to-detector distance within a few minutes as well as the possibility to implement a broad range of sample environments such as an imaging ellipsometer or a sputter deposition chamber highlight the exceptional modularity of the beamline. The selected examples illustrate the high potential of the MiNaXS beamline in terms of high-flux X-ray scattering beamline for *in situ* and time-resolved investigations of kinetic phenomena involved in growth processes and transport phenomena.

## Footnotes

1 SESO X Ray Beam Monitor, http://www.seso.com/pub/X Ray Beam Monitor.pdf (last access: 16 December 2011).

2 Roper Scientific Germany Quad-RO 4096 four-port readout X-ray camera, http://www.roperscientific.de/QuadRo.html (last access: 16 December 2011).

3 DECTRIS PILATUS 300k,  http://www.dectris.com/pilatus-300k.html (last access: 16 December 2011).

4 SII Vortex-EM, http://www.siintusa.com/details.php?products_id=4 (last access: 16 December 2011).

5 DESY Detector Loan Pool, http://hasylab.desy.de/instrumentation/detectors/detector__loan_pool/ (last access: 16 December 2011).
